# The Effect of *Aspergillus Thermomutatus Chrysovirus* 1 on the Biology of Three *Aspergillus* Species

**DOI:** 10.3390/v10100539

**Published:** 2018-10-02

**Authors:** Mahjoub A. Ejmal, David J. Holland, Robin M. MacDiarmid, Michael N. Pearson

**Affiliations:** 1 School of Biological Sciences, the University of Auckland, Auckland 1142, New Zealand; mejm158@aucklanduni.ac.nz (M.A.E.); robin.macdiarmid@plantandfood.co.nz (R.M.M.); 2 Infectious Diseases Unit, Division of Medicine, Middlemore Hospital, Auckland 1640, New Zealand; David.Holland@middlemore.co.nz; 3 Plant and Food Research, Auckland 1142, New Zealand

**Keywords:** chrysovirus, mycovirus, *Aspergillus*, *A. fumigatus*, *A. nidulans*, *A. niger*, *A. thermomutatus*, biocontrol

## Abstract

This study determined the effects of *Aspergillus thermomutatus* chrysovirus 1 (AthCV1), isolated from *Aspergillus thermomutatus*, on *A. fumigatus*, *A. nidulans* and *A. niger*. Protoplasts of virus-free isolates of *A. fumigatus*, *A. nidulans* and *A. niger* were transfected with purified AthCV1 particles and the phenotype, growth and sporulation of the isogenic AthCV1-free and AthCV1-infected lines assessed at 20 °C and 37 °C and gene expression data collected at 37 °C. AthCV1-free and AthCV1-infected *A. fumigatus* produced only conidia at both temperatures but more than ten-fold reduced compared to the AthCV1-infected line. Conidiation was also significantly reduced in infected lines of *A. nidulans* and *A. niger* at 37 °C. AthCV1-infected lines of *A. thermomutatus* and *A. nidulans* produced large numbers of ascospores at both temperatures, whereas the AthCV1-free line of the former did not produce ascospores. AthCV1-infected lines of all species developed sectoring phenotypes with sclerotia produced in aconidial sectors of *A. niger* at 37 °C. AthCV1 was detected in 18% of sclerotia produced by AthCV1-infected *A. niger* and 31% of ascospores from AthCV1-infected *A. nidulans.* Transcriptome analysis of the naturally AthCV1-infected *A. thermomutatus* and the three AthCV1-transfected *Aspergillus* species showed altered gene expression as a result of AthCV1-infection. The results demonstrate that AthCV1 can infect a range of *Aspergillus* species resulting in reduced sporulation, a potentially useful attribute for a biological control agent.

## 1. Introduction

Mycoviruses have been reported in a wide variety of fungi [1]. In nature, mycoviruses may be vertically transmitted through both asexual and sexual spores, although the rate of transmission varies depending upon the fungal species, the virus and the growth conditions [2]. Horizontal transmission is typically via hyphal anastomosis which is often limited by hyphal incompatibility, even within a species [3], so consequently most mycoviruses have a narrow natural host range. However, there is growing evidence that extracellular transmission may occur [4,5,6] potentially providing a mechanism for inter-species transmission, although to date this has only been demonstrated in a few instances [4,5,6].

While the vast majority of mycoviruses appear to have little or no obvious impact on their host, there are reports of significant changes in the phenotype and growth characteristics of some fungi due to viral infection. These alterations include reduction in growth and virulence of plant pathogenic fungi [7,8,9,10,11], cytological alterations of cellular organelles [12], changes in pigmentation [13] and enzymatic activities [14], enabling the host fungus to confer heat tolerance to its host plant [15] and production of killer toxins [16,17]. Moreover, dsRNAs of viral origin are known to be interferon inducers [18].

Like many mycoviruses, most chrysoviruses are associated with latent infections of their fungal hosts [19]. However, there are reports of negative impacts on the fungal host attributed to chrysovirus infection, including: attenuated growth, hypovirulence and sector formation caused by *Botryosphaeria dothidea* chrysovirus 1 [20]; weakened growth, altered pigmentation and abnormal hyphal aggregation induced by *Magnaporthe oryzae* chrysovirus 1-A [21]; the absence of aerial hyphae formation and subsequent conidiophore development with impaired mycelial growth and albino hyphae produced due to *Magnaporthe oryzae* chrysovirus 1-B infection [22]; hypovirulence of the host fungal strain caused by *Botryosphaeria dothidea* chrysovirus 1 [23]; aconidial sectoring, decreased pigmentation and reduced biomass induced by *Aspergillus fumigatus* chrysovirus [24]; and aconidial sectoring, low spore production and reduced radial growth caused by *A. niger* chrysovirus [25].

The effects of mycovirus infection on gene expression have been studied for some fungi. In a study of four different *Fusarium graminearum* viruses [26] a hypovirus (FgV1), a chrysovirus (FgV2), a totivirus (FgV3) and a partitivirus (FgV4) were individually used to infect *Fusarium graminearum* strain PH-1 via protoplast fusion. An RNA-Seq-based transcriptome analysis of each of the virus-infected PH-1 cultures showed that all four mycoviruses affected the transcriptome profiles and each mycovirus regulated the expression of a totally different set of host genes. However, transcriptome profiles of the naturally infected *Fusarium graminearum* strains do not appear to have been included in that assessment. Also, Sun et al. [27] showed that levels of Mycoreovirus 1-Cp9B21 genomic dsRNA increased when the virus was co-infecting *Cryphonectria parasitica* together with the Cryphonectria hypovirus 1-EP713.

We previously reported [28] sector formation, with noticeable changes in colony texture and color, plus changes in sporulation rate in cultures of *A. thermomutatus* (isolate Ath-1) infected with *Aspergillus thermomutatus* chrysovirus 1 (AthCV1). This paper presents the results of a subsequent study to investigate the ability of AthCV1 to infect other *Aspergillus* species (*A. fumigatus*, *A. niger* and *A. nidulans*) and the biological and molecular impacts of AthCV1 on these species.

## 2. Materials and Methods

### 2.1. Source of Aspergillus Isolates

An isolate of *A. thermomutatus* infected with AthCV1 was used as a source of virus particles for protoplast transfection of *A. fumigatus* (Afu-13, dsRNA-free), *A. niger* (Ang-9, dsRNA-free) and *A. nidulans* (And-1, dsRNA-free). Clinical isolates of Afu-13 and Ang-9 were provided by Dr David Holland, Clinical Director Infection Services, Middlemore Hospital, Auckland, New Zealand and And-1 was provided by Wendy McKinney, Mycology Reference Laboratory, Auckland Hospital. Cultures were maintained on Potato Dextrose Agar (PDA, DifcoTM, BD Diagnostics, Heidelberg, Germany) in the dark at 37 °C and 20 °C.

### 2.2. Virus Purification and Detection

AthCV1 was purified as follows: Virus-infected *A. thermomutatus* was grown in Yeast Extract Peptone Dextrose broth (YPD Broth, Yeast Extract 5 g/L, Microbiological peptone 3 g/L and Dextrose 10 g/L) and incubated on a shaking incubator at 180 rpm in the dark for 2 days. Approximately 10 g of fungal mycelium was harvested on a filter paper using vacuum filtration, ground to a fine powder in liquid nitrogen and transferred to a 50 mL Falcon tube containing 20 mL of sodium phosphate buffer (SPB) (0.1 M, pH 7.0) and 10 mL chloroform. The mixture was incubated for 30 min on ice on an orbital shaker, at 230 rpm and then centrifuged at 10,000× *g* for 30 min at 4 °C. The upper aqueous phase was centrifuged at 120,000× *g* for 2 h at 4 °C and the resultant pellet was re-suspended in 1 mL SPB (0.02 M pH 7.0) for 4 h at 4 °C. The suspension was clarified by centrifugation at 10,000× *g* for 20 min at 4 °C, the supernatant centrifuged at 120,000× *g* for 2 h at 4 °C and the pellet was re-suspended in 0.5 mL SPB (0.02 M, pH 7.0) overnight at 4 °C. Following centrifugation at 10,000× *g* for 20 min a 50-µL drop of the supernatant was negatively stained with 2% uranyl acetate (pH 4.0) and observed for virus particles using a Phillips CM12 TEM. The virus was detected by virus-specific reverse transcription polymerase chain reaction (RT-PCR), using virus specific primers that amplified a 639 bp product from the coat protein (genome segment 2), as described by Ejmal et al. [28].

### 2.3. Protoplast Preparation and Virus Transfection

Protoplast preparation and virus transfection were undertaken as described by Ejmal et al. [28]. Briefly, 5 g of one-day old mycelium, grown in YPD Broth, was washed once with sterile distilled water and once with protoplast buffer (0.8 M MgSO_4_·7H_2_O, 0.2M C_6_H_5_Na_3_O_7_·2H_2_O, pH 5.5). The mycelium was then coarsely chopped and transferred to a flask containing 17 mL protoplast buffer. Three mL of filter sterilized Novozyme buffer (1 M Sorbitol, 50 mM Sodium citrate, pH 5.8) containing 200 mg of Lysing Enzymes from *Trichoderma harzianum* (Sigma-Aldrich, St. Louis, MO, USA) was added to the mycelial suspension, which was incubated for 4 h at 28°C in a shaker at 85 rpm. Protoplasts were passed through a 75-µm strainer and collected in a 50-mL tube containing 30 mL KC buffer (0.6 M KCl and 50 mM CaCl_2_) and centrifuged at 4000× *g* for 10 min. The protoplast pellet was then washed twice with 10 mL sorbitol-Tris-calcium chloride (STC) buffer (1M Sorbitol, 50 mM Tris, pH 8 and 50 mM CaCl_2_.2H_2_O) and centrifuged at 4000× *g* for 10 min before being was re-suspended in 0.5 mL STC and kept on ice. For transfection, 130 µL PEG 4000 (60% in sterile water) was mixed with 70 µL potassium chloride-Tris-Calcium Chloride (KTC) buffer (1.8 M KCl, 150 mM Tris pH 8, 150 mM CaCl_2_) and added to a tube containing 200 µL of purified virus particle suspension and 5 µL 0.05 mM spermidine (Sigma-Aldrich, St. Louis, MO, USA). A 200-µL aliquot of the protoplast suspension was then added to the suspension and mixed by twirling the tube for 10 s before it was incubated on ice for 30 min. Following incubation, a mix of 200 µL of polyethylene glycol (PEG) 4000 and 100 µL KTC was added to the previous suspension and gently twirled again. Following incubation at room temperature for 20 min, 40-µL aliquots of the mixture were added to 5 mL warm agar medium (Stabilized Minimal Medium (SMM) containing 0.7% agar), gently mixed and spread on SMM plates, which were parafilm sealed and incubated at 37 °C. Individual colonies were picked off, grown on fresh PDA plates and sub-cultured three times, at weekly intervals, then checked for the presence of the virus by RT-PCR as described by Ejmal et al. [28]. As negative controls, three plates were spread with a protoplast suspension lacking virus particles, to test protoplast viability. In addition, three plates were spread with no protoplasts in the transfection suspension to test for possible mycelial contamination in the virus particle suspension. As a general contamination check, three plates containing only SMM media were included.

### 2.4. Quantifying the Biological Impacts of AthCV1 Infection on Three Aspergillus Species

Sporulation rate comparisons were conducted at 20 °C to represent environmental temperature and 37 °C to represent human body temperature. Five virus-free and five virus-infected lines were grown from single spores, inoculated at the edge of PDA plates (9 cm diameter) and incubated in the dark until the mycelium reached the opposite side of the plate. To harvest conidiospores, the plates were washed with 40 mL aqueous 0.05% Tween 80, which was then filtered through cheesecloth and centrifuged at 8000× *g* for 10 min. The spores were re-suspended in 10 mL distilled water before being counted in a Neubauer chamber, as described by Aneja [29].

For linear growth comparison, five replicates of isogenic virus-free and virus-infected single spore isolates were individually inoculated at the edge of PDA plates and grown at 37 °C and at 20 °C. The growth was measured every 24 h until the mycelium reached the far edge of the plate. At the completion of each experiment cultures were tested for the presence of the virus using RT-PCR as described by Ejmal et al. [28].

To compare biomass production five isogenic virus-free and virus-infected single spore isolates, of each species, were grown at 37 °C and at 20 °C in the dark. Plugs of the resultant mycelium were individually transferred to conical flasks containing 200 mL YPD Broth and incubated with shaking at 180 rpm, in the dark. The resultant mycelium was vacuum filtered and 100 mg of each sample retained for virus screening by one-step RT-PCR by Ejmal et al. [28]. The remainder was dried at 90 °C for 72 h before weighing. Data were analyzed by an independent samples *t*-test, using SPSS version 21 (IBM SPSS statistics, Armonk, NY, USA).

### 2.5. AthCV1 Transmission Through Ascospores and Sclerotia

AthCV1 transmission through *A. nidulans* ascospores was tested as follows: First ascospores were processed to eliminate contamination from mycelium and conidia, based on the methods of O’Gorman et al. [30] and Girardin et al. [31] and individually germinated on PDA. Once the single ascospore cultures had produced sufficient mycelium they were screened for the presence of AthCV-by RT-PCR using virus specific primers as described by Ejmal et al. [28]. To test for AthCV1 in sclerotia produced by *A. niger*, the sclerotia were isolated from cultures on PDA plates incubated at 37 °C for 2 weeks in the dark, according to the method of Utkhede and Rahe [32], with minor modifications as follows: Sclerotia were picked from the agar plates with forceps and placed in a 100 mL beaker containing 75 mL sterile water, filtered through three layers of cheesecloth, washed with sterile water and surface sterilized with 0.25% sodium hypochlorite for 2 min. The sclerotia were then immediately passed through 3 layers cheesecloth and washed again with 500 mL sterile water. One hundred sclerotia were individually inoculated to PDA plates and incubated at 37 °C for 2 weeks in the dark before the resultant mycelium was screened for AthCV1 infection using one-step RT-PCR as described by Ejmal et al. [28].

### 2.6. The Effects of AthCV1 on Gene Expression

Both AthCV1-infected and uninfected cultures of *A. thermomutatus*, *A. fumigatus*, *A. niger* and *A. nidulans* were grown on sterile cellophane film overlaid on PDA media. The cultures were incubated at 37 °C for 5 days in the dark, as described by Zhang et al. [33], to provide growth conditions similar to those used for growth and sporulation experiments. To minimize the effects of variability between cultures, three replicates for each treatment were pooled before total RNA was extracted. Thirty milligrams of mycelium from each of three plates was harvested and combined in a 2-mL Eppendorf tube and total RNA extracted using a Spectrum Plant Total RNA Kit (Sigma-Aldrich, St. Louis, MO, USA), as described by the manufacturer. A 0.1 volume of 3 M sodium acetate, pH 5.2 and two volumes of 100% ethanol were added to each extract to precipitate the RNA, which was sent on ice to Macrogen Inc. (Soeul, Korea), for RNA sequencing. An Agilent Technologies 2100 Bioanalyzer 2100 Bioanalyzer (Agilent Technologies, Santa Clara, CA, USA) was used to measure RNA quality and quantity of the original samples and confirm an RNA integrity number (RIN) of 8 or greater. Fragmentation was performed on RNA samples before cDNA synthesis and the cleaved RNA fragments primed with random hexamers. First strand cDNA was transcribed using reverse transcriptase according to the Truseq RNA sample preparation V2 guide (Illumina, San Diego, CA, USA). The second cDNA strand synthesis was performed using DNA polymerase I in the presence of RNase H. Adapters were ligated to the DNA and PCR amplification performed to selectively enrich DNA fragments with adapter molecules on both ends and to amplify the amount of DNA in the library. PCR amplification was conducted using a PCR Primer Cocktail which anneals to the ends of the adapters. An Agilent Technologies 2100 Bioanalyzer (Agilent Technologies, Santa Clara, CA, USA) with a DNA 1000 chip was used to verify the size of PCR-enriched fragments and for library quantification qPCR was used, according to the Illumina qPCR Quantification Protocol Guide (# 11322363) (Illumina, San Diego, CA, USA). Indexed DNA libraries were normalized to 10 nM in the Diluted Cluster Template (DCT) plate and then pooled in equal volumes in the Pooled DCT plate. Sequencing was conducted using the Hiseq2000 platform (Illumina, San Diego, CA, USA) which generated reads of 100 bp × 2 (paired-end) with a total run output of 35–40 Gb. The FastQC quality control tool [34] was used to provide quality control checks on each sequence data file.

For *A. fumigatus*, *A. niger* and *A. nidulans*, where a reference genome was available (fungi.ensembl.org/index.html), the Tuxedo protocol [35] was used. The 100-bp paired-end output reads data files for each treatment (5 GB each) were uploaded individually to the Galaxy-qld platform (galaxy-qld.genome.edu.au) and Tophat2, version 0.6 [36] used to align the sequences (both forward and reverse reads). Each BAM file, containing accepted hits produced by Tophat2, was then assembled using Cufflinks software version 0.0.7 [37]. Cuffmerge software version 0.0.6 [37] was then used to merge all the assembled transcripts together in one output GTF file and Cuffdiff software version 0.0.7 [37] used to identify possible significant changes in transcript expression between AthCV1-negative and positive lines. Following that, identifiers of the differentially expressed genes were used to search for their gene ontology annotations in *Aspergillus* genome databases (www.aspergillusgenome.org), Ensemble Fungi database (fungi.ensembl.org/index.html) and the UniProt Knowledgebase (www.uniprot.org).

For *A. thermomutatus*, where no reference genome was available, the Trinity *de novo* transcriptome assembly software (version 0.0.2) [38,39] was used to create a full-length transcriptome which can be processed by Tophat and cufflinks. The Trinity read normalization tool was used to reduce coverage of highly covered areas and then the Galaxy “concatenate datasets” tool (version 1.0.0) used to combine all normalized forward and reverse reads, for each treatment. The combined reads were then used to produce a fasta file that contained all possible assembled transcripts from both AthCV1-positive and AthCV1-negative samples. The resultant transcriptome was used for the Tuxedo protocol, as described above. From the Cuffdiff output, gene loci identifiers (e.g., comp0_c0_seq1) were used to retrieve their relevant transcripts and the DNA sequences used as queries for nucleotide BLASTn searches in GenBank, to find the closest available gene sequences, which were used to search for gene ontology annotations.

## 3. Results

### 3.1. Transfection of Aspergillus Species

**Figure 1:**
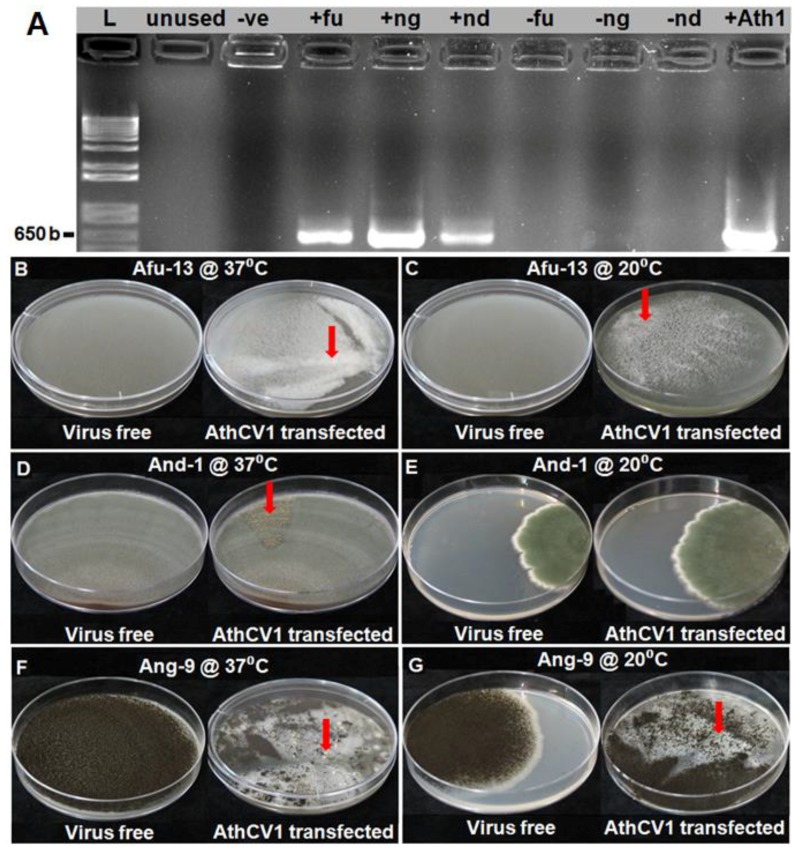
Effects of AthCV1 infection on the colony morphology of *Aspergillus* species. (**A**) polymerase chain reaction (PCR) assay showing successful AthCV1 transfection to different virus-free *Aspergillus* species; reverse transcription PCR, lane L = 1 kb plus DNA ladder; -Ve= PCR negative control (RNA sample was replaced with ultrapure water), +Ath1 = AthCV1 +ve control; +fu = AthCV1 transfected *A. fumigatus* (Afu-13); +ng = AthCV1 transfected *A. niger* (Ang-9); +nd = AthCV1 transfected *A. nidulans* (And-1); –fu, –ng and –nd = original virus-free isolates of *A. fumigatus* (Afu-13), *A. niger* (Ang-9) and *A. nidulans* (And-1), respectively. (**B**) Afu-13 grown at 37 °C; normal growth in the virus-free isolate, sectors formed in isogenic AthCV1-transfected line (arrow). (**C**) Afu-13 grown at 20 °C; normal growth in the virus-free isolate, sectors formed in isogenic AthCV1-transfected line (arrow). (**D**) And-1 grown at 37 °C; normal growth in the virus-free isolate, ascospore-rich sectors formed in isogenic AthCV1-transfected line (arrow). (**E**) And-1 grown at 37 °C; normal growth in both virus-free and AthCV1-transfected lines. (**F**) Ang-9 grown at 37 °C; normal growth in the virus-free isolate, conidial-free sectors with sclerotia formed (arrow) in AthCV1-transfected line. (**G**) Ang-9 grown at 20 °C; normal growth in the virus-free isolate, conidial- and sclerotia-free sectors in AthCV1-transfected line (arrow).

AthCV1-specific RT-PCR, conducted on the third serial subcultures of *A. fumigatus* (Afu-13), *A. niger* (Ang-9) and *A. nidulans* (And-1), confirmed that all three species were successfully transfected with AthCV1 purified particles (Figure 1A).

### 3.2. Impact of AthCV1 on Sporulation of Aspergillus Spp.

The results for the effects of AthCV1 on sporulation of *A. fumigatus* (Afu-13), *A. niger* (Ang-9) and *A. nidulans* (And-1) at 37 °C and 20 °C, together with the results for *A. thermomutatus* previously published [28] are presented in Table 1. In *A. fumigatus*, AthCV1 infection significantly reduced (*p* ≤ 0.05) asexual sporulation at both temperatures, with the effects being more extreme at 37 °C than at 20 °C. As would be expected there was no sexual reproduction observed in either the AthCV1-free or AthCV1-infected treatments at either temperature as *A. fumigatus* is a heterothallic species. In the homothallic species *A. nidulans* conidial production was significantly reduced (*p* ≤ 0.05) in the AthCV1-infected line at 37 °C but not at 20 °C, while there was a significant increase (*p* ≤ 0.05) in the number of ascospores produced by the AthCV1-infected line at both 37 °C and 20 °C. In *A. niger* asexual sporulation was significantly decreased (*p* ≤ 0.05) in the AthCV1-infected line at 37 °C while there was no significant difference between AthCV1-free and infected lines at 20 °C. *A. niger* also formed sclerotia in the AthCV1-Infected line at 37 °C but not at 20 °C. There was no sexual reproduction in *A. niger* cultures at either temperature.

### 3.3. Effects of AthCV1 on Aspergillus Growth and Morphology

A summary of the effects of AthCV1 on the growth and the cultural characteristics of the *Aspergillus* species at 37 °C and 20 °C, together with the results for *A. thermomutatus* previously published [28], are presented in Table 1. The radial growth of *A. fumigatus* on PDA plates was significantly greater (*p* ≤ 0.05) in AthCV1-infected cultures at 20 °C but not at 37 °C. In contrast, there was a significant decrease in mycelial dry weight associated with AthCV1 infection at 37 °C but not at 20 °C. In addition, while the AthCV1-free culture produced a uniform grey-colored mycelium, the mycelium of the AthCV1-infected line formed small white sectors that subsequently merged together. This was more prominent at 37 °C than at 20 °C, eventually covering most of the plate (Figure 1B,C). In *A. nidulans* there was a significant decrease in radial mycelial growth of the AthCV1-infected line at 20 °C while at 37 °C there was no difference in growth between AthCV1-free and AthCV1-infected lines. Mycelial dry weight was lower in AthCV1-infected cultures at 37 °C but there was no change in mycelial dry weight at 20 °C. A sectoring phenotype was observed in AthCV1-infected cultures grown at 37 °C which were very rich in sexual fruiting bodies (Figure 1D) but not in those grown at 20 °C (Figure 1E). In *A. niger* there was a significant reduction of radial mycelial growth on PDA plates in infected cultures at 37°C but no difference in mycelial growth at 20 °C. There was a significant increase in mycelial dry weight in the AthCV1-infected line grown at 20 °C but no difference between virus-infected and virus-free at 37 °C. Sectors with a conidia-free phenotype occurred in the AthCV1-positive culture at both 37 °C and 20 °C (Figure 1F,G). At 37 °C sclerotia were formed in these conidia-free sectors (Figure 1F) but no sclerotia were formed in cultures grown at 20 °C (Figure 1G).

### 3.4. Transmission of AthCV1 Through Ascospores and Sclerotia

In AthCV1 infected cultures, ascospores were produced by *A. nidulans* but not in *A. fumigatus* or *A. niger*. Of the single ascospore cultures grown at 37 °C, 31 of 100 were AthCV1-infected, while from the culture grown at 20 °C, 34 of 100 ascospores were infected. Sclerotia were produced in AthCV1 infected cultures of *A. niger* but not *A. nidulans* or *A. fumigatus.* AthCV1 was detected in 18 of 100 sclerotia produced by *A. niger* grown at 37 °C.

### 3.5. Changes in Gene Expression Associated with AthCV1 Infection

**Figure 2:**
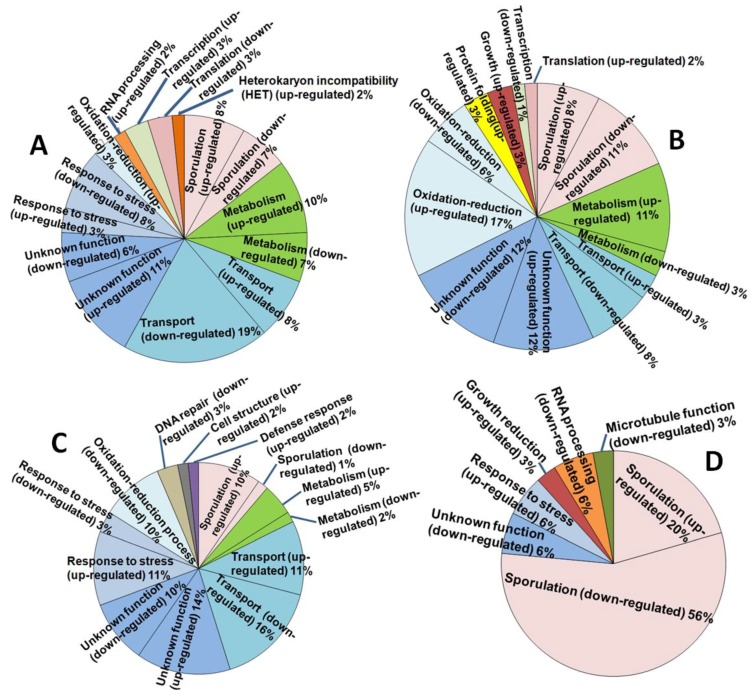
Differentially expressed genes in AthCV1-infected *Aspergillus* species and their functional analysis. (**A**) *A. thermomutatus*; (**B**) *A. niger*; (**C**) *A. fumigatus*; (**D**) *A. nidulans*.

The quality and quantity check performed on the original total RNA samples indicated that all of the samples were of acceptable quality (RIN = 6.5–7.6). The average RNA size was *c*. 260–280 bp, of which 126 bp represented the Illumina sequencing adapters, yielding a gene sequence fragment of *c*. 140–160 bp. FastQC indicated an acceptable Phred quality score of ≥20 over all samples. FastQC also confirmed that the encoding format of the data in the RNA-seq files was in “Sanger/Illumina 1.9” format as required for the Galaxy platform. De novo transcriptome assembly for *A. thermomutatus*, for which no genome was available by the time of the study, produced a fasta file containing 44430 sequences. FPKM (Fragments Per Kilobase of transcript, per Million mapped reads) results indicated that for AthCV1-infected cultures some genes were highly upregulated while others showed downregulation or were undetectable (i.e., showed a zero FPKM value), compared with the isogenic virus-free line. These included genes related to fungal sporulation (Table 2). The number of differentially expressed genes (≥ 5-fold change or undetectable in one treatment) were 62 each in *A. thermomutatus* and in *A. fumigatus*, 65 in *A. niger* and 34 in *A. nidulans*. The functional analysis of these genes is shown in Figure 2.

As shown in Table 3, the effect of AthCV1 infection on gene expression varied between the different *Aspergillus* species. Some genes showed expression change in only one species, such as the transcription factor gene *steA* (AN2290), for which changes were observed only in *A. niger*. For other genes, the variation was just in the degree of expression, as for the orthologue of the heat shock protein gene, *hsp30*/*hsp42* (AN5781). In other instances, the difference was in the direction of regulation, such as the internal alternative NADH dehydrogenase *ndiA* (AN10660), which was completely repressed in *A. nidulans* but upregulated by 25-fold in *A. fumigatus* and by 18-fold in *A. niger*. The orthologue of this gene in *N. crassa* has an NADPH dehydrogenase activity and a role in spore germination with mitochondrial inner membrane localization (Table 2). In yeast (*S. cerevisiae*) overexpression of the orthologue (*ndi-1*) of this gene (*ndiA*) can result in apoptosis-like cell death, which is associated with an increase in the production of reactive oxygen species (ROS) in mitochondria [42] and the transcription factor *nosA*/AN1848, which in the AthCV1-infected lines was reduced 326-fold in *A. niger*, 20-fold in *A. nidulans* and 2-fold in *A. fumigatus* but was unchanged in *A. thermomutatus*.

## 4. Discussion

Mycoviruses have received much attention in relation to their potential as biological control agents for harmful fungi. Two necessary properties of biocontrol agents are (i) the ability to reduce the growth and virulence of the target fungus and (ii) rapid and efficient transmission of the virus from the inoculum to the target fungal population. Hyder et al. [43] found that the effects of a virus isolate on its fungal host can differ depending upon the strain of the infected fungus and that the extent of those effects can vary according to the environment and ecological conditions. Similarly, Vainio et al. [44] found that conditions (such as temperature) can alter virus impact on its fungal host. Consequently, the development of mycoviruses as effective biological control agents requires an understanding of both the viruses and their interaction with their fungal hosts and environment.

Vegetative incompatibility is a major barrier to studying the effects of viruses on a range of fungal hosts and their use in the field, as it can be a serious barrier to horizontal virus transmission between different fungal species and even different strains of the same fungal species. However, in vitro, this obstacle may be overcome by the use of protoplast fusion [45,46,47,48] or transfection of protoplasts with purified virus particles [48,49]. The latter technique has been successfully used for the transfection of virus-free *A. fumigatus* protoplasts with *Aspergillus fumigatus* tetramycovirus (AfuTmV-1) [50] and *Aspergillus fumigatus chrysovirus* (AfuCV) [24], as well as species of other fungal genera, including *Botryosphaeria dothidea* with *Botryosphaeria dothidea chrysovirus* 1 (BdCV1) [20], *Rhizoctonia solani* with *Rhizoctonia solani partitivirus* 2 [51], *Cryphonectria parasitica* with a reovirus from *C. parasitica* [52], *Rosellinia necatrix* with *Rosellinia necatrix partitivirus* (RnPV1) [53] and *Rosellinia necatrix mycoreovirus 3* (RnMYRV-3) [54]. In the current study, we successfully transfected *A. fumigatus*, *A. niger* and *A. nidulans* with AthCV1 particles from naturally infected *A. thermomutatus*, to produce isogenic virus-free and virus-infected lines, in order to evaluate the effects of the virus on these fungal hosts.

The impact of AthCV1 infection on mycelial growth on PDA plates varied from no change at either 37 °C or 20 °C for *A. thermomutatus*, to significantly reduced growth for *A. niger* at 37 °C and *A. nidulans* at 20 °C and a significant increase in the growth for *A. fumigatus* at 20 °C. An increase in linear growth due to virus infection was previously reported by Nuss [55] where *C. parasitica* (strain Euro7) cultures infected with *Cryphonectria hypovirus* 1 (CHV-1/Euro7) showed faster growth than the isogenic virus-free isolate. However, linear growth on agar plates does not necessarily correlate with total biomass production, as seen in our experiments for *A. fumigatus*, *A. nidulans* and *A. niger* (Table 1), or with growth and/or pathogenicity in vivo [56,57].

In the current investigation, sectoring (areas with different appearance) were observed in the virus-infected cultures of all four *Aspergillus* species. Sectoring has been observed in association with a range of mycoviruses including, BdCV1 [20], *Helminthosporium victoriae* co-infected with the totivirus, *Helminthosporium victoriae virus* 190S (HvV190S) and the chrysovirus, *Helminthosporium victoriae virus* 145s (HvV145S) [56]. In a study by Bhatti et al. [24], *A. fumigatus* formed non-sporulating sectors when infected with *A. fumigatus partitivirus* 1 (AfuPV-1) or with AfuCV. However, the basis of fungal sectoring can have a number of underlying causes other than virus infection.

Although virus infection resulted in changes in sporulation and phenotype of all three species, the different *Aspergillus* species did not respond to AthCV1 infection in the same way. The response was often temperature dependent; *A. fumigatus* reacted consistently at both temperatures with significantly reduced conidial production (Table 1), whereas *A. thermomutatus* showed a significant increase in conidia at 37 °C but produced significantly fewer conidia at 20 °C (Table 1). In the AthCV1-infected *A. nidulans* (homothallic), ascospore production was significantly increased at both 37 °C (90%) and 20 °C (170%) compared to uninfected. *A. thermomutatus* (also homothallic) produced ascospores only when infected with AthCV1 (Table 1), although the number produced at 20 °C was about half that produced at 37 °C (Table 1). Individual isolates of *A. niger* and *A. fumigatus* do not produce ascospores in culture as these species are not homothallic.

Since vertical transmission and dissemination of virus infection is largely dependent on spores, the rate of infection in spores is an important factor. For many fungi, virus transmission through conidia is often high (<100%) [47,58,59], while transmission via ascospores is generally less efficient [60,61]. Coenen et al. [62] concluded that vertical transfer of dsRNA viruses in *A. nidulans* did not occur through ascospores resulting in the exclusion of viruses in the fungal population, presumably because vegetative incompatible groups formed as a result of sexual recombination in the successive generations. However, other studies have shown varying degrees of virus transmission through ascospores, including transmission of *Botrytis Virus* X (BVX) through ascospores in *Botrytis cinerea* [63], dsRNA virus particles in *S. cerevisiae* [64] and two different dsRNA mycoviruses in *Fusarium graminearum* [58]. In the current study AthCV1 was detected in 34% and 31% of ascospores at 20 °C and 37 °C, respectively.

The production of large numbers of ascospores in AthCV-1 infected *A. thermomutatus,* compared to no ascospores in the virus-free line and a significant increase in ascospore production in *A. nidulans*, together with the decrease in conidial production, could be the result of physiological stress due to AthCV1 infection, as stress is often a trigger for a change from asexual to sexual reproduction in fungi [65]. Therefore, AthCV1 could potentially increase sexual diversity of the fungus by stimulating ascospore production, creating the opportunity for increased gene sharing and increased sexual diversity. In addition, sclerotia, which are often produced under nutrient-depleted conditions (e.g., in old cultures) and are thought essential for sexual reproduction in *Aspergillus* sections *Flavi* [40] and *Circumdati* [66] and *A. japonicas* [67], were produced in *A. niger* at 37 °C. Frisvad et al. [66] were the first to report the production of sclerotia by *A. niger* although most of the *A. niger* strains used in their study failed to produce sclerotia despite the multiple attempts to enhance the process. It is possible that mycovirus infection affected their two strains that produced sclerotia, however the strains used in their study were screened for the presence of mycoviruses. In summary, the findings in this study indicate that AthCV1 infection enhances sexual sporulation in *A. nidulans* and *A. thermomutatus* and possibly also in *A. niger* where sclerotia were produced at 37 °C.

In the current study variability in the nature of the sectoring phenotype and differences in sporulation, mycelial growth diameter and mycelial dry weight associated with AthCV1 infection in the four different *Aspergillus* species demonstrate that the effect of AthCV1 is both species dependent and temperature dependent.

Given that AthCV1 has significant effects on asexual and sexual spore reproduction in some *Aspergillus* species, knowledge of which metabolic pathways are affected might possibly be exploited for better control of the fungus. Consequently, preliminary gene expression data, based on pooled samples of three replicate cultures for each treatment, were obtained to identify changes in the expression of known reproduction-related genes as a starting point for future, more extensive studies. In response to AthCV1 infection changes in expression were detected in a total of 223 different genes, across the four *Aspergillus* species examined. Genes specifically related to sporulation and/or reproduction numbered 54, those showing greater than five-fold changes (either + or −) are presented in Table 2.

An important family of genes for sexual reproduction in fungi are the Mating Type (MAT) genes which enable homothallic fungi to switch their mating type. In *A. nidulans*, Paoletti et al. [68] have shown that overexpression of *MAT2* represses MAT1 expression and vice versa. The current findings reflect this with the two homothallic species *A. thermomutatus* and *A. nidulans* where *MAT1*/NFIA_071100 and *MAT1*/AN2755 were downregulated in the AthCV1-infected treatment by 8-fold and 16-fold respectively whereas, *MAT2* was upregulated by 4-fold in the AthCV1-infected *A. nidulans* and expressed in the AthCV1-infected *A. thermomutatus* but not in the virus-free line.

In AthCV1-infected *A. nidulans,* other highly upregulated genes that contribute to the increase in sexual sporulation include: *gprk*/AN7795 [40] (39-fold increase) and AN3695 (www.aspergillusgenome.org) (13-fold), *fhbA*/AN7169 [69] (12-fold). Genes that were undetected and whose repression might have contributed to the high reduction in conidiation include *mst1*/AN5674 (positive regulation of asexual development and negative regulation of sexual development) [70] and *nudF*/AN6197 (positive regulation of conidiation and ascospore production) [71]. In addition, genes that might have a negative impact on sexual reproduction if overexpressed and were somewhat downregulated included *cryA*/AN0387 (37-fold decrease) that codes for a blue light- and UVA-sensing cryptochrome that represses sexual development by regulating other regulators such as VeA, NsdD and RosA [72] and *hogA*/AN1017 (6-fold decrease) which is a putative mitogen-activated protein kinase (MAPK), is highly up-regulated under osmotic stress conditions required for sexual development and sporulation [73]. In AthCV1-infected *A. nidulans* conidiation was hugely reduced and this seems consistent with the increased number of ascospores, as it is known that ascospore production can inhibit conidiation and vice versa [40]. Changes in expression of genes known to be involved in the reduction of conidiation included the downregulation of *conF*/AN8640 (21-fold reduction) which contributes in conidia germination and in the protection of conidia against desiccation [41], *noxR*/AN6046 (13-fold reduction) which is important for conidiophore development and conidia production [74] and the putative septin B, *aspB*/AN6688 (12-fold reduction), which plays a role in growth emergence and conidiation [75,76].

In AthCV1-infected *A. thermomutatus* there was a 7-fold increase in expression of *csnB*/NFIA_070550, which is essential for cleistothecia and ascospore formation [77,78]. The expression of *laeA*/NFIA_010750, an orthologue of the global regulator of secondary metabolism (*laeA*/AN0807), was downregulated by 1913-fold. This gene coordinates asexual development in response to light, is involved in the regulation of secondary metabolism and is required for the formation of Hulle cells [79,80,81,82,83]. The NirA-dependent flavohemoprotein gene (*fhbA*/NFIA_030070), which is involved in the positive regulation of sterigmatocystin production and in the negative regulation of sexual sporulation [69], was downregulated by 100-fold.

Genes of *A. niger* that were affected and possibly contributed to the huge decrease of conidiation in the AthCV1-infected treatment at 37 °C included: the putative C2H2 transcription factor (*flbC*/An02g05420) which showed a 31-fold downregulation and has a predicted role in conidiation and is expressed in germinating conidia [84,85,86]; An04g07400 (326-fold reduction) an orthologue of *adv-1* in *N. crassa* which is required for normal sexual and asexual development [87]; *ppoA*/An04g05880 (20-fold decrease in expression), the miss expression of the *A. nidulans* orthologue *ppoA*/AN1967 leads to a decreased level of conidiation (http://www.aspergillusgenome.org); and An05g00480 (5-fold reduction) which is expressed in germinating conidia and has a predicted role in positive regulation of conidiophore development and conidium formation (www.aspergillusgenome.org). In addition, An14g02540, the orthologue of the sclerotium regulator in *A. oryzae* (*sclR*/AO090011000215), that encodes a transcription factor with a role in hyphal morphology and the promotion of sclerotial production [88], was upregulated by 11-fold in the AthCV1-infected treatment. This is possibly an indication that the production of sclerotia was also related to stress, as they are survival structures typically produced in response to environmental stress [89]. Also, An18g06650, which has a strong similarity to *A. nidulans* heat shock protein 30 (*hsp30*/AN2530) [86], showed a 157-fold increase in expression. An orthologue of this gene in *A. fumigatus*, Afu3g14540 (*hsp30*), has been found to be expressed in a high abundance in conidia and is known to increase in response to the antifungal amphotericin B and to hydrogen peroxide [90,91].

Transcriptome analysis of the heterothallic *A. fumigatus* revealed the greatest upregulation (6427-fold increase) for Afu1g04410, which has no known function and the greatest downregulation (18094-fold reduction) for the DNA N-glycosylase, putative (Afu7g05320), the orthologue of which in *S. cerevisiae* has a predicted role in oxidized purine nucleobase lesion DNA N-glycosylase activity and molecular function in DNA damage repair (www.uniprot.org). Other *A. fumigatus* genes that showed altered transcription levels due to AthCV1 infection were: *ppoA*/Afu4g10770 (7-fold reduction), an orthologue of *A. nidulans ppoA*/AN1967, which is involved in the response to oxidative stress, negative regulation of sexual sporulation and positive regulation asexual sporulation [92] and Afu1g09750 (38-fold reduction) which is reported to be enriched in conidia compared to mycelium of *A. fumigatus* [90]; and Afu1g14520 (25-fold increase) which is orthologous to *N. crassa* (*ndi-1*) and has a predicted role in oxidation-reduction process and spore germination [93]. In addition, conidial pigmentation biosynthesis genes that were highly expressed in AthCV1-infected *A. fumigatus* included: Scytalone dehydratase *arp1*/Afu2g17580 (9-fold increase), which is involved in conidial pigment biosynthesis and for which mutants display increased C3 complement binding [94,95]; Afu2g17550/*ayg1* which is involved in conidial pigment biosynthesis with a role in polyketide shortening and melanin biosynthesis in *Aspergillus fumigatus* [94,96]; *pksP*/Afu2g17600 (12-fold increase) which is involved in biosynthesis of the conidial pigment and asexual spore wall assembly [97]; *arp2*/Afu2g17560 (16-fold increase) which is involved in conidial pigment biosynthesis and expression of conidia-enriched protein [90,94]. The latter three genes may be important virulence factors in the establishment of infection as mutations in these genes showed increased virulence and failed to inhibit phagolysosome acidification in their insect host *Galleria mellonella* [98].

In response to AthCV1 infection all four of the *Aspergillus* species included in this study exhibited changes in phenotype (Table 1) and gene expression (Table 3). For some factors, similar changes were observed in two or more of the *Aspergillus* species while for others the different species varied in their response. This is similar to the findings of Hyder et al. [43] who studied two viruses of the wood decay fungus *Heterobasidion* and reported that (i) a specific virus strain can cause different effects on different *Heterobasidion* strains and (ii) the impact of a single virus strain on a certain *Heterobasidion* isolate can differ according to the changes in environmental conditions. The diversity in response of the four *Aspergillus* species to AthCV1 supports the proposition that even if a particular mycovirus is found to have a hypovirulent effect on a certain fungal isolate, it does not necessarily follow that it will have the same effect on other isolates of the same or closely related species. Consequently, any evaluation of the potential of mycoviruses as biological control agents should be conducted on a range of fungal isolates in a range of environmental conditions. In the current study, differences in the expression of the internal alternative NADH dehydrogenase NdiA (AN10660) in different *Aspergillus* species is probably a good example of this phenomenon as it was upregulated by 25-fold in *A. fumigatus* and by 18-fold in *A. niger* but entirely repressed in *A. nidulans*.

In-vitro transfection can be used to experimentally extend the host range of a mycovirus in order to investigate the potential effects on new fungal hosts. This ability to overcome the barriers caused by hyphal incompatibility and genetic diversity may, if it results in stable transfected lines, extend the application of mycoviruses, such as AthCV1, for fungal disease control. However, it is important to understand the wider implications of specific phenotypic changes (e.g., spore production), observed under controlled experimental conditions, to the epidemiology of the fungus in the environment. For example, while the low conidiation rate of AthCV1-infected *A. fumigatus* and *A. niger* may reduce the ability of the fungus to spread and compete with uninfected fungal isolates in the field and thereby limit the impact of AthCV1 on *Aspergillus*, the high number of ascospores produced by the AthCV1-positive *A. nidulans* and *A. thermomutatus* and the enhancement of sclerotia production in *A. niger* may improve the survival of these fungi, as these structures typically resist adverse environmental changes better than conidiospores. Moreover, the presence of virus could directly or indirectly, increase diversity of *Aspergillus* by stimulating sexual reproduction and possibly increase the percentage of vegetatively compatible fungal strains allowing virus spread between them.

The use of mycoviruses as effective biological control agents requires consideration of multiple factors (host, virus and environment). The first objective is to find a virus that is capable of inducing serious impact on its fungal host. Once such a virus is identified, intensive research on how and when to use the virus is required, for example, direct application to the site of infection in the case of chestnut blight control [99]. A major constraint on the use of mycoviruses is that natural horizontal virus spread typically requires hyphal anastomosis, which is limited by genetically controlled hyphal incompatibility. Consequently, the direct use of mycoviruses as biological control agents, especially in a clinical context, is very challenging. However, an understanding of the molecular nature of the hypovirulence caused by a particular mycovirus may enable the development of a more direct approach such as targeting the expression of certain genes by pharmaceuticals that mimic the mechanism of hypovirulence.

## Figures and Tables

**Table 1:** Summary of the effects of AthCV1 on four *Aspergillus* species.

		*A. thermomutatus*		*A. fumigatus*		*A. niger*		*A. nidulans*	
Property	Growth Temp	Virus-Free	AthCV1 Infected	Virus-Free	AthCV1 Infected	Virus-Free	AthCV1 Infected	Virus-Free	AthCV1 Infected
Conidia production (per plate)	37 °C	2.1 (± 0.36) × 10^5^	2.0 (± 0.10) × 10^6^ *	3.8 (± 0.42) × 10^8^	1.6 (± 0.12) × 10^7^ *	1.8 (± 0.12) × 10^8^	2.0 (± 0.14) × 10^7^ *	2.2 (± 0.62) × 10^8^	9.5 (± 0.79) × 10^7^ *
	20 °C	2.4 (± 0.36) × 10^6^	2.1 (± 0.24) × 10^5^ *	1.5 (± 0.21) × 10^8^	3.6 (± 0.31) × 10^7^ *	2.2 (± 0.26) × 10^8^	1.9 (± 0.24) × 10^8^	1.4 (± 0.16) × 10^8^	1.4 (± 0.12) × 10^8^
Ascospore production (per plate)	37 °C	0	7.2 (± 0.73) × 10^5^ *	0	0	0	0	1.1 (± 0.10) × 10^6^	2.1 (± 0.42) × 10^6^ *
	20 °C	0	3.6 (± 0.37) × 10^5^ *	0	0	0	0	2.9 (± 0.22) × 10^5^	8.0 (± 0.55) × 10^5^ *
Sclerotia production (per plate)	37 °C	0	0	0	0	0	54 (± 4.2) *	0	0
	20 °C	0	0	0	0	0	0	0	0
Growth (mm) ^1^	37 °C	74 (± 0.32)	75 (± 1.00)	72 (± 0.37)	73 (± 1.00)	77 (± 0.20)	67 (± 0.68) *	76 (± 0.49)	75 (± 0.71)
	20 °C	76 (± 0.32)	75 (± 0.32)	67 (± 0.32)	72 (± 0.20) *	77 (± 0.60)	67 (± 0.74)	33 (± 0.45)	30 (± 0.66) *
Fungal Biomass ^2^ (g dry wt)	37 °C	1.25 (± 0.012)	1.28 (± 0.009)	1.30 (± 0.008)	1.23 (± 0.008) *	1.25 (± 0.023)	1.30 (± 0.007)	1.36 (± 0.021)	1.12 (± 0.005) *
	20 °C	1.13 (± 0.011)	1.13 (± 0.009)	1.15 (± 0.011)	1.13 (± 0.007)	1.09 (± 0.004)	1.12 (± 0.003) *	1.12 (± 0.016)	1.12 (± 0.010)
Sector formation	37 °C		Creamy, rough ascospore-rich sectors		Clear, elongated sectors in the grey mycelium		Conidia-free sectors with no pigmentation		Ascospore-rich sectors in the green mycelium
	20 °C		Creamy, rough ascospore-rich sectors		Clear, elongated sectors in the grey mycelium		Conidia-free sectors with no pigmentation		No sector formation
Pigment change	37 °C		Creamy sectors		Sectors lack pigmentation		Sectors lack pigmentation		No change
	20 °C		Creamy sectors		Sectors lack pigmentation		Sectors lack pigmentation		No change

Data analyzed by independent samples *t*-test using SPSS version 21, SEs in brackets. * = significant difference (*p* < 0.05) between virus-free and virus infected treatments.^1^ Since growth was measured to the edge of the petri dish the growth period differed for the different species: at 37 °C *A. thermomutatus* = 6 days, *A. fumigatus* = 6 days, *A. niger* = 13 days, *A. nidulans* = 13 days; at 20 °C *A. thermomutatus* = 15 days, *A. fumigatus* = 24 days, *A. niger* = 29 days, *A. nidulans* = 24 days. ^2^ Growth in liquid culture was measured after 4 days at 37 °C and 15 days at 20 °C.

**Table 2:** Regulation of sporulation-related genes in AthCV1-infected *Aspergillus species* compared with their isogenic AthCV1-free line. Genes showing a five-fold or greater difference in expression between infected and non-infected lines are presented in the table.

Gene ID	Verified and Predicted Gene and Function ^◊^ retrieved from www.aspergillusgenome.org, www.uniprot.org and fungi.ensembl.org/index.html	Fold Change
***A. thermomutatus*^∆^**		
NFIA_070550	*csnB*; orthologue(s) subunit2 of the COP9 signalosome; required for formation of cleistothecia	7 ↑
NFIA_010750	*laeA*; orthologue (*laeA*/AN0807, *A. nidulans*) coordinates asexual development in response to light, involved in the regulation of secondary metabolism and required for the formation Hulle cells.	1913 ↓
NFIA_030070	*fhbA*; flavohemoprotein, regulates sexual sporulation and sterigmatocystin production	100 ↓
NFIA_071100	*MAT1*; positive regulation of mating type specific transcription, DNA-templated	35 ↓
***A. fumigatus***		
Afu1g14520	Pyridine nucleotide-disulphide oxidoreductase; NADPH dehydrogenase activity, spore germination	25 ↑
Afu2g17560	Hydroxynaphthalene reductase arp2; involved in conidial pigment biosynthesis; conidia-enriched protein	16 ↑
Afu2g17600	Conidial pigment polyketide synthase PksP/Alb1; conidial pigment biosynthesis; conidia wall assembly	13 ↑
Afu2g17550	Heptaketide hydrolase ayg1; conidial pigment; polyketide shortening; conidia-enriched protein	13 ↑
Afu2g17580	Scytalone dehydratase arp1; conidial pigment biosynthesis; conidia formation and sporulation	9 ↑
Afu1g09750	Aldehyde reductase (AKR1), putative; conidia-enriched protein	38 ↓
Afu4g10770	*ppoA*; response to oxidative stress; orthologue (AN1967/ppoA) negative regulation of sexual sporulation and positive regulation asexual sporulation.	7 ↓
***A. niger***		
An18g06650	Orthologue (Afu3g14540, *A. fumigatus*), 30-kilodalton heat shock protein; conidia-enriched protein	157 ↑
An01g12490	Orthologue (*Neurospora crassa*) has NADPH dehydrogenase activity, role in spore germination	18 ↑
An14g02460	*fhbA*; orthologue in *A. nidulans* regulation of sexual sporulation and sterigmatocystin biosynthetic process	12 ↑
An04g07400	Putative C6 zinc finger transcription factor; orthologue (AN1848/*nosA*, *A. nidulans*) positive regulation of sexual development; orthologue (*adv-1*) in *N. crassa* has predicted role in conidium formation and hyphal growth.	326 ↓
An12g00710	Orthologue (AN9121/*esdC*, *A. nidulans*); negative regulation of conidiation and positive regulation of sexual sporulation	200 ↓
An02g05420	*flbC*; putative C2H2 transcription factor; predicted role in conidiation; expressed in germinating conidia	31 ↓
An04g05880	*ppoA*; response to oxidative stress; orthologue (AN1967/*ppoA*) negative regulation of sexual sporulation and positive regulation of asexual sporulation.	20 ↓
An01g04830	*flbD*; Myb-like DNA-binding protein; positive regulation of conidiation; expressed in germinating conidia	12 ↓
An17g01580	Orthologue (AN2290/steA, *A. nidulans*) has negative regulation of transcription by RNA polymerase II promoter; regulation of secondary metabolite biosynthetic process; sporocarp development involved in sexual reproduction	5 ↓
An05g00480	*stuA*; positive regulation of conidium formation and conidiophore development	5 ↓
***A. nidulans***		
AN7795	*gprK*; positive regulation of sexual sporulation [40]	39 ↑
AN3387	*gprD*; Putative G-protein coupled receptor; Deletion of *gprD* resulted in delayed conidial germination and enhanced sexual development	16 ↑
AN3695	Putative anthranilate synthase with a predicted role in aromatic amino acid biosynthesis and cleistothecium development	13 ↑
AN7169	*fhbA*; flavohemoprotein; sterigmatocystin biosynthetic process and regulates sexual development	12 ↑
AN5844	Controls conidia germination and adjusts cellular substances which protect conidia against dryness [41]	7 ↑
AN5046	*anisin-1*; asexual sporulation, response to oxidative stress and defense response	7 ↑
AN5156	*pho80*; overexpression decreases conidiation and increases formation of cleistothecia	6 ↑
AN3148	PH domain protein; have role in ascospore wall assembly, ascospore-type prospore membrane assembly	44 ↓
AN0387	*cryA*; negative regulation of cleistothecium development	37 ↓
AN8640	*conF*; Conidiation protein Con-6, putative; contributes in conidia germination and desiccation resistance	21 ↓
AN1848	*nosA*; Zinc(II)2Cys6 putative transcription factor involved in the regulation of sexual development	20 ↓
AN2755	*MAT1*; regulator of sexual development; acts with Mat2 HMG domain protein	16 ↓
AN6046	*noxR*; P67phox regulatory subunit homolog; required for normal sexual and asexual development	13 ↓
AN6688	*aspB*; conidiophore development and hyphal growth	12 ↓
AN4163	*cpcB*; required for sexual development; positive regulation of cleistothecium development	11 ↓
AN4351	*palA*; pH-response regulator protein palA; orthologue in *Saccharomyces cerevisiae* (RIM20) role in sporulation resulting in formation of a cellular spore	6 ↓
AN1017	*hOGA*; Putative mitogen-activated protein kinase; required for sexual development and sporulation	6 ↓
AN7553	*devR*; Basic helix-loop-helix transcription factor required for normal conidiophore development	5 ↓
AN10306	*candA-N*; role in sexual development, secondary metabolism and light control of asexual development	5 ↓
AN2458	*candA-C*; role in sexual development, secondary metabolism and light control of asexual development	5 ↓

∆ = due to the absence of *A. thermomutatus* genome, gene orthologues of *Neosartorya fischeri* (closest BLAST hits) were used. ◊ = function is for the gene if available or for *A. nidulans* gene orthologue. ↑ = up-regulated in the AthCV1-infected line; ↓ = down-regulated in the AthCV1-infected line.

**Table 3:** Major changes in the expression of gene orthologues of AthCV1-infected *Aspergillus* species compared to their isogenic AthCV1-free lines.

Fold Change in AthCV1-infected Samples					Gene and Function From www.uniport.org & www.aspergillusgenome.org
Gene ID *A. nidulans*	*A. thermomutatus*	*A. fumigatus*	*A. nidulans*	*A. niger*	
AN10660	-	25	-	18	*ndiA*; Pyridine nucleotide-disulphide oxidoreductase, putative; NADPH dehydrogenase activity and spore germination.
AN2755	−35	-	−16	-	*MAT1*: Alpha-domain mating-type protein; regulator of sexual development; acts with Mat2 HMG domain protein.
AN1652	-	−5	-	-	*sebA*; C2H2 transcription factor; required for virulence; response to oxidative stress and heat shock.
AN5781	-	31	6	157	Heat shock protein Hsp30, transcript increase during the unfolded-protein response; palA-dependent expression independent of pH
AN2458	3	-	−5	-	*canadA-C*; orthologue(s) N-terminal subunit of Cand1; sexual development, secondary metabolism and light control of asexual development.
AN10306	5	-	−5	3	*candA-N*; role in sexual development and secondary metabolism.
AN10049	-	9	−4	-	*mdpB*; Probable scytalone dehydratase; involved in conidial pigment biosynthesis, conidium formation and sporulation.
AN5836	-	-	−4	−5	*stuA*; Positive regulation of asexual sporulation and conidiophore development.
AN1848	-	−2	−20	−326	*nosA*; Zinc(II)2Cys6 putative transcription factor; positive regulation of sexual development.
AN5156	−2	-	6	-	Pho80-like cyclin; overexpression decreases conidiation and increases cleistothecia.
AN7169	−100	4	12	12	*fhbA*; Flavohemoglobin, negative regulation of sexual sporulation and positive regulation of sterigmatocystin biosynthesis.
AN0807	−1913	−2	-	−4	*laeA*; Methyltransferase-domain protein; self-methylates; coordinates asexual development in response to light; regulates secondary metabolism and is required for Hulle cell formation.
AN0387	-	2	−37	-	*cryA*: Negative regulation of cleistothecium development.
AN4783	7	-	−2	-	*csnB*; COP9 signalosome subunit 2 (CsnB) formation of cleistothecia.
AN2421	4	−3	-	−31	*flbC*: regulation of conidium formation and spore germination.
AN7553	-	−3	−5	-	*devR*; Basic helix-loop-helix transcription factor; conidiophore development.
AN5893	−5	2	-	-	*flbA*; Developmental regulator FlbA, conidiophore development and asexual sporulation.
AN0082	−6	−2	−2	−2	*phnA*; Phosducin, putative; regulates sporulation
AN3387	-	2	16	−2	*gprD*: Deletion of gprD resulted in delayed conidial germination and enhanced sexual development.
AN0279	-	−2	2	−12	*flbD*: Myb-like DNA-binding protein; positive regulation of conidiation.
AN2290	-	-	-	−5	*steA*; Sexual development transcription factor SteA; Required for cleistothecial development and ascosporogenesis. Not required for conidiation.
AN0170	-	-	-	−24	*trxA*; Thioredoxin, required for conidiation; expression upregulated after exposure to farnesol.
AN9121	2	-	-	−200	*esdC*; sexual development protein, involved in early sexual development and regulated by VeA and FlbA.
AN4163	-	2	−11	-	*cpcB*: G-protein complex beta subunit CpcB; positive regulation of cleistothecium development.
AN1017	2	−2	−6	2	*hogA*; Putative mitogen-activated protein kinase; required for sexual development and sporulation.
AN7795	-	-	39	-	*gprK*; positive regulation of sexual sporulation [40].
**Up-regulated**		**Down-regulated**			
Colour	Fold change	Colour	Fold change		
	<5		<5		
	5 to 20		5 to 20		
	21 to 100		21 to 100		
	101 to 500		101 to 500		
	> 500		> 500		
